# Lack of interleukin-13 receptor α1 delays the loss of dopaminergic neurons during chronic stress

**DOI:** 10.1186/s12974-017-0862-1

**Published:** 2017-04-21

**Authors:** Simone Mori, Shuei Sugama, William Nguyen, Tatiana Michel, M. Germana Sanna, Manuel Sanchez-Alavez, Rigo Cintron-Colon, Gianluca Moroncini, Yoshihiko Kakinuma, Pamela Maher, Bruno Conti

**Affiliations:** 1 Department of Molecular Medicine, The Scripps Research Institute, 10550 N. Torrey Pines Road, La Jolla, CA 92037 USA; 2 Department of Physiology, Nippon Medical School, Tokyo, 113-8602 Japan; 3 Department of Infection and Immunity, Luxembourg Institute of Health, Esch-sur-Alzette, L-4354 Luxembourg; 4 Department of Neuroscience, The Scripps Research Institute, 10550 N. Torrey Pines Road, La Jolla, CA 92037 USA; 5 Dipartimento di Scienze Cliniche e Molecolari, Università Politecnica delle Marche, 60020 Ancona, Italy; 6 Cellular Neurobiology Laboratory, Salk Institute for Biological Studies, La Jolla, CA 92307 USA; 7 Dorris Neuroscience Center, The Scripps Research Institute, 10550 N. Torrey Pines Road, La Jolla, CA 92037 USA

**Keywords:** Stress, Interleukin, Parkinson’s disease, Neuroinflammation, Microglia, Oxidative stress

## Abstract

**Background:**

The majority of Parkinson’s disease (PD) cases are sporadic and idiopathic suggesting that this neurodegenerative disorder is the result of both environmental and genetic factors. Stress and neuroinflammation are among the factors being investigated for their possible contributions to PD. Experiments in rodents showed that severe chronic stress can reduce the number of dopaminergic neurons in the substantia nigra pars compacta (SNc); the same cells that are lost in PD. These actions are at least in part mediated by increased oxidative stress. Here, we tested the hypothesis that the interleukin-13 receptor alpha 1 (IL-13Rα1), a cytokine receptor whose activation increases the vulnerability of dopaminergic neurons to oxidative damage, participates in the stress-dependent damage of these neurons.

**Methods:**

Mice were subject to daily sessions of 8 h (acute) stress for 16 weeks (5 days a week), a procedure previously showed to induce loss of dopaminergic neurons in the SNc. The source and the kinetics of interleukin-13 (IL-13), the endogenous ligand of IL-13Rα1, were evaluated 0, 1, 3, 6, and 8 h and at 16 weeks of stress. Identification of IL-13 producing cell-type was performed by immunofluorescent and by in situ hybridization experiments. Markers of oxidative stress, microglia activation, and the number of dopaminergic neurons in IL-13Rα1 knock-out animals (*Il13ra1* ^*Y/***−**^) and their wild-type littermates (*Il13ra1* ^*Y/+*^) were evaluated at 16 weeks of stress and at 20 weeks, following a 4 week non-stressed period and compared to non-stressed mice.

**Results:**

IL-13 was expressed in microglial cells within the SN and in a fraction of the tyrosine hydroxylase-positive neurons in the SNc. IL-13 levels were elevated during daily stress and peaked at 6 h. 16 weeks of chronic restraint stress significantly reduced the number of SNc dopaminergic neurons in *Il13ra1* ^*Y/+*^mice. Neuronal loss at 16 weeks was significantly lower in *Il13ra1* ^*Y*/−^ mice. However, the loss of dopaminergic neurons measured at 20 weeks, after 4 weeks of non-stress following the 16 weeks of stress, was similar in *Il13ra1* ^*Y/+*^ and *Il13ra1* ^*Y*/−^ mice.

**Conclusions:**

IL-13, a cytokine previously demonstrated to increase the susceptibility of SNc dopaminergic neurons to oxidative stress, is elevated in the SN by restraint stress. Lack of IL-13Rα1 did not prevent nor halted but delayed neuronal loss in the mouse model of chronic restraint stress. IL-13/IL-13Rα1 may represent a target to reduce the rate of DA neuronal loss that can occur during severe chronic restraint stress.

**Electronic supplementary material:**

The online version of this article (doi:10.1186/s12974-017-0862-1) contains supplementary material, which is available to authorized users.

## Background

Parkinson’s disease (PD) is the second most common neurodegenerative disorder affecting approximately 1% of the population over the age of 60 [1]. Clinical symptoms include tremors, rigidity, and postural instability which are believed to arise primarily from the progressive reduction of dopamine signaling in the basal ganglia resulting from the loss of dopaminergic (DA) neurons in the substantia nigra pars compacta (SNc). Although several genes have been demonstrated to contribute to familial PD, the majority of PD cases are sporadic and idiopathic, with environmental factors thought to contribute to the development of the disease [2]. Stressful psychological events and neuroinflammation are among the environmental factors being investigated in PD research.

The contribution of stress to neurodegeneration was studied extensively in the hippocampus where the hormone glucocorticoids were demonstrated to mediate neuronal loss [3–5]. Correlative and experimental evidences also suggested that stress may contribute to loss of dopaminergic neurons and thus contribute to the etiology or to the progression of PD [6–14]. Several studies also showed that different types of stressor including restraint, [15–18], prenatal [19], dental disharmony [20], and cold stress [21], can lead to the activation of microglial-increasing reactive oxygen species (ROS) and interleukins [22] creating a neuroinflammatory environment that is believed to contribute to PD [23–25].

In an effort to elucidate the possible contribution of neuroinflammation to PD and how its supposedly non-specific action could lead to a preferential loss of DA neurons, we previously showed that this is mediated, at least in part, by activation of the receptor alpha 1 of interleukin-13 (IL-13Rα1) [26]. IL-13Rα1 is known for its peripheral role of mediating allergic inflammation and atopy, but it is also expressed centrally in DA neurons of the SNc and the VTA where its activation increased cellular susceptibility to oxidative stress [26]. Interestingly, *Il13ra1* is localized at position Xq24 of the human X chromosome, within a region containing the PARK 12 locus associated with PD.

Preliminary studies showed that restraint stress elevated the level of IL-13 in the CNS [27]. Here, we tested the hypothesis that activation of IL-13Rα1 contributes to the loss of DA neurons occurring in a mouse model of severe chronic restraint stress (RS) consisting of daily 8 h long RS sessions for 5 days a week for 16 weeks [11]. We determined the central cellular source of IL-13 that measured the kinetics of its production and the effects that stress had on microglial activation and oxidative damage during the daily session of 8 h RS and at 16 weeks. The number of DA neurons in the SNc in mice null for IL-13Rα1 (*Il13ra1* ^*Y/−*^) and their wild-type littermates (*Il13ra1* ^*Y/+*^) was determined and compared at 16 or at 20 weeks, 4 weeks after termination of stress.

## Methods

### Animals and tissue harvesting

Mouse husbandry and procedures were approved and performed under the guidelines of the Institutional Animal Care and Use Committee of The Scripps Research Institute and by the Institutional Animal Care and Use Committee of Nippon Medical School and were performed in accordance with the National Institute of Health Guide for the Care and Use of Laboratory Animals; animal suffering and sacrifice were minimized. All experiments were carried out on 3–6-month-old male mice null for IL-13Rα1 (*Il13ra1* ^*Y−*^) and wild-type littermates (*Il13ra1* ^*Y/+*^) on a C57BL/6 background [28]. Animals were housed in a room maintained at 20–22 °C, on a 12-h light/12-h dark with food and water provided ad libitum. For tissue collection, animals were deeply anaesthetized using 4–5% isoflurane and subsequently perfused with 0.1 M phosphate buffer for real-time PCR analysis or with 0.1 M phosphate buffer followed by 4% paraformaldehyde (PFA) for histological analysis. After overnight post-fixation in 4% PFA, the brains to be used for histological analysis were cryoprotected in 30% sucrose then frozen at −80 °C. The brains were sectioned using a Leica cryostat to produce 35 μm slices for both immunohistochemistry and immunofluorescence.

### Stress experiment

**Fig. 1:**
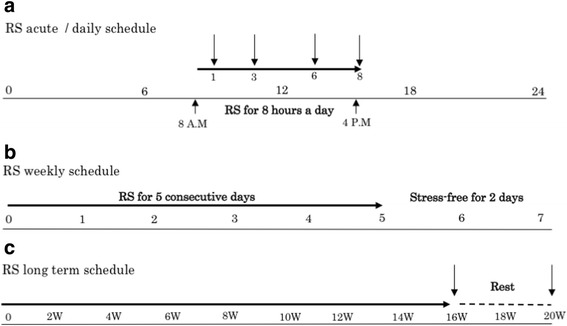
Experimental restraint stress paradigms. Schematic representation of stress regimen and experimental design. **a** Daily sessions of 8 h RS were performed from 8 AM to 4 PM. Levels of IL-13 transcripts were determined at 1, 3, 6, and 8 h and the neuroinflammatory response. **b** Weekly schedule of RS consisted 8 h RS/day for 5 consecutive days per week followed by 2 days of non-stress. **c** Severe chronic RS consisted of the weekly RS schedule for a maximum up to was applied for 16 weeks. Distinct groups of animals were maintained in non-stressed conditions for four additional weeks following last session of stress before tissue was collected for analysis

Restraint stress (RS) was performed as previously described [11] on age-matched *Il13ra1* ^*Y/−*^ and *Il13ra1* ^*Y/+*^ mice. Briefly, animals were placed in ventilated plexiglass restrainers that prevented the mice from turning without causing any pain for a maximum of eight consecutive hours per day. Chronic stress was achieved by performing this acute stress procedure daily for 5 days a week for a maximum of 16 weeks; a regimen previously shown by us to induce loss of DA neurons in the SNc [11] (Fig. 1a, b). Tissues were also collected at the end of the last 8-h session at 16 weeks RS or at the same time of day after 4 additional weeks after termination of stress (week 20) (Fig. 1c).

### Immunohistochemistry

Immunofluorescence microscopy was performed on 35-μm brain slices containing the region of interest (see Additional file 1: Figure S1a). The following primary antibodies were used: rabbit-anti Iba-1 (1:500, Wako); rabbit anti-GFAP (1:250, Thermo Fisher); mouse monoclonal anti-NeuN (1:250, Millipore); goat anti-IL-13 (1:50, R&D system), rabbit anti-3-Nitrotyrosine (3-NT, 1:200, Millipore). After washing, a combination of secondary antibodies was used for detection: 488 Alexa goat anti-rabbit, 594 Alexa goat anti-mouse IgG1 (Invitrogen). Slides were counterstained with DAPI and were cover-slipped in Fluoromount. Digital images were taken using a Zeiss LSM 710 laser scanning confocal microscope (LSCM). Tyrosine Hydroxylase DAB Immunohistochemical analysis was performed using rabbit anti-Tyrosine Hydroxylase, (1:10000; Millipore), incubated with a biotinylated goat-anti rabbit antibody (1:400; Vector Labs) for 1 h at RT and then stained with Vectastain ABC Kit (Standard) and DAB Peroxidase Substrate (Vector Labs). For cresyl violet (CV) staining (Nissl), adjacent sections were mounted on positive charged slides and air-dried. The slides were soaked in CV solution (0.25% cresyl violet and 0.3% acetic acid) for 15 min (warmed at 50 °C), were dehydrated with alcohol and xylene, and were cover-slipped with Vectamount.

### Cell counting

Cell counting was performed by unbiased stereology. Briefly, digital images of TH- and CV-positive cells were acquired on the SNc at ×50 magnification on an Olympus microscope fitted with a video camera (Olympus, Tokyo, Japan). Counting frames (100 × 100 μm) were generated using MCID image analysis software (Imaging Research Inc., Ontario, Canada) and were systemically scanned over the outlined SNc using a motorized stage. Neurons were counted when they appeared within a square (50 × 50 μm) of the counting frame but were not in contact with the left or bottom border. This procedure was carried out on every four sections at a periodicity of 140 μm through the SNc. Finally, total SNc neuron number was calculated as the product of the neuron density and the volume of the SNc as previously reported [29, 30]. To count the number of double-positive-labeled cells (IL13-green) and (Iba-1, GFAP, NeuN, or Tyrosine Hydroxilase-red), we used region of interest analysis macros in Image Pro Premier (Media Cybernetics) [28]. Digital images were taken using a Zeiss LSM 710 laser scanning confocal microscope LSCM. A maximum intensity projection (MIP) was created of each multi-sectioned reconstruction of the double-positive-labeled cells using the Zen software. For further processing, the MIPs of each sample were imported into Image Pro Premier (Media Cybernetics) where the region of the SN was defined (outline) to create a mask of the whole region of interest. Four counting frames (100 × 100 μm) were generated using Image Pro Premier Software for each MIP image. The number of red, green, and total nuclear count (DAPI) in each frame was counted with the exclusion of those not in contact with the left or bottom border. These results were represented as number of cell/total nuclear count extracted from the total area for each fluorescent-labeled population. Double-positive populations were defined by scoring, outlined as a region of interest, all of one population (signal Red-TH), and obtaining from the software how much of the other (green-IL-13) resides within the red-positive region of interest. 3-NT fluorescence intensity arbitrary units (a.u.) were calculated by dividing the detected optical density by area of the region of interest.

### Tissue processing for protein quantification and semi-quantitative PCR

Real-time PCR and IL-13 ELISA were performed on SN samples from IL-13Rα1 wild-type mice. Animals (6–7 per group) were perfused intracardially with PBS at different time points of acute RS (3, 6, and 8 h), and the SN was rapidly dissected and flash frozen. Total RNA was isolated using a RNA extraction kit (NucleoSpin RNA Plus, Macherey-Nagel #740984.250) according to the manufacturer’s protocol. After eluting the RNA product with RNase/DNase-free water, the concentration and purity of the RNA was determined using a NanoDrop ND-1000 spectrophotometer. Total RNA was reverse transcribed into cDNA using a reverse transcription cDNA synthesis kit (Maxima cDNA Synthesis Kit, Thermo Fisher, #FERK1642) per the manufacturer’s protocol. The cDNA templates were amplified via PCR using the Taq polymerase enzyme (PowerUP Sybr Master Mix, Life Technologies, #A25777) along with mouse IL-13-specific primers obtained from Qiagen RT2 qPCR Primer Assay, (PPM03021B). IL-13Rα1 primers used as follows: FW 5′-cct tgc ttc tgt ggt agt aga g-3′ and RW 5′- aaa ggg cca gga gga aat ac-3′. A Bio-Rad CFX 384 PCR System was used to perform the qPCR reactions. Data were analyzed using the ∆∆Ct method with the levels of the gene of interest normalized to the beta Actin housekeeping gene. For protein extraction, SN samples were homogenized in 400 μl of lysis buffer (N-PER™ Neuronal Protein Extraction Reagent, Thermo Fisher, plus 1 protease inhibitor cocktail tablet) and then were centrifuged at 14,000*g* for 30 min at 4 °C. The supernatants were collected, and protein concentrations were determined by Pierce BCA Protein Assay Kit (Thermo Fisher Scientific, USA). Samples were analyzed in a ELISA for mouse IL-13 (R&D) according to the manufacturer’s instruction, and results normalized for total protein (pg IL-13/mg total protein).

### Statistical analysis

One-factor ANOVA followed by a Bonferroni post hoc test was used to determine statistical significance (*p < 0.05, **p < 0.01; ***p < 0.001) using GraphPad Prism Software (La Jolla, CA, USA).

## Results

### IL-13 is increased by acute RS in the substantia nigra

**Fig. 2:**
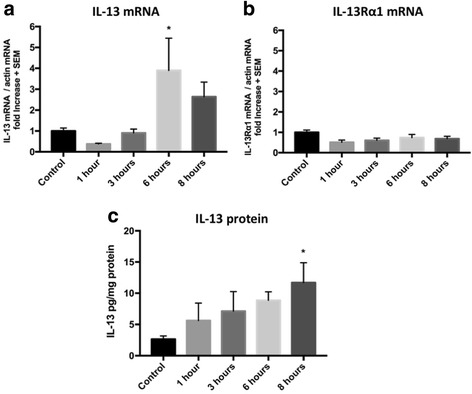
Acute restraint stress induces central production of IL-13 in the substantia nigra. **a** Semi-quantitative RT-PCR performed on RNA extracted from the SN of mice treated with acute RS showed that compared with the level of control animals, arbitrarily fixed to 1 (*n* = 8), levels were 0.37 ± 0.032, *p* > 0.99; 0.90 ± 0.186, *p* > 0.99; 3.89 ± 1.545, *p* = 0.04 and 2.63 ± 0.704, *p* = 0.74 at 1, 3, 6, and 8 h, respectively, *n* = 6. **b** Semi-quantitative RT-PCR assessing expression of IL-13Rα1 on same samples described in figure A shows no difference in all time points compared to control condition (*p* > 0.99 at control, 1, 3, 6, and 8 h). **c** Protein quantification in the SN shows upregulation of IL-13 during restraint stress (IL*-*13 in pg/mg total protein: 2.644 ± 0.50 control; 5.607 ± 2.793 at 1 h RS, *p* > 0.99;7.128 ± 3.123, at 3 h, *p* = 0.679; 8.857 ± 1.361 at 6 h RS, *p* = 0.1612, and 11.7 ± 3.195 at 8 h RS, *p* = 0.0342)

IL-13Rα1 is expressed in dopaminergic neurons where its activation during neuroinflammation increases their vulnerability to oxidative stress in the SNc [26]. We previously demonstrated that repetitive sessions of acute daily of 8 h RS can eventually reduce the number of DA neurons in the SNc [11]. To determine whether the daily session of acute stress would modify the level of IL-13, we measured IL-13 mRNA, immunoreactivity, and protein level in the SN of animals restrained for 0, 1, 3, 6, or 8 h (Fig. 2). Semi-quantitative RT-PCR analysis revealed that RS elevated the IL-13 mRNA. The increase was the highest and most significant at 6 h of RS with fold change compared to non-stressed animals being 3.89 ± 1.545, *n* = 6, *p* = 0.04. Fold difference compared to control (arbitrarily assigned the value of 1) did not reach statistical significance at the other time-point tested (0.37 ± 0.032, *n* = 6, *p* > 0.99 at 1 h RS; 0.90 ± 0.186, *n* = 6, *p* > 0.99 at 3 h RS; 2.63 ± 0.704, *p* = 0.74 at 8 h RS) (Fig. 2a). The levels of IL-13Rα1 mRNA were also determined (Fig. 2b) with no alterations on the mRNA levels of the IL-13 receptor at all time points. ELISA demonstrated that RS also elevated the level of IL-13 protein and that such increase was statistically significant at 8 h when the fold change compared to non-stressed animals was of a similar order of magnitude as the transcript at 6 h (IL-13 in pg/mg total protein: 2.644 ± 0.50 control; 5.607 ± 2.793 at 1 h RS, NS; 7.128 ± 3.123, at 3 h, NS; 8.857 ± 1.361 at 6 h RS, NS, and 11.7 ± 3.195 at 8 h RS, *p* < 0.05) (Fig. 2c).

**Fig. 3:**
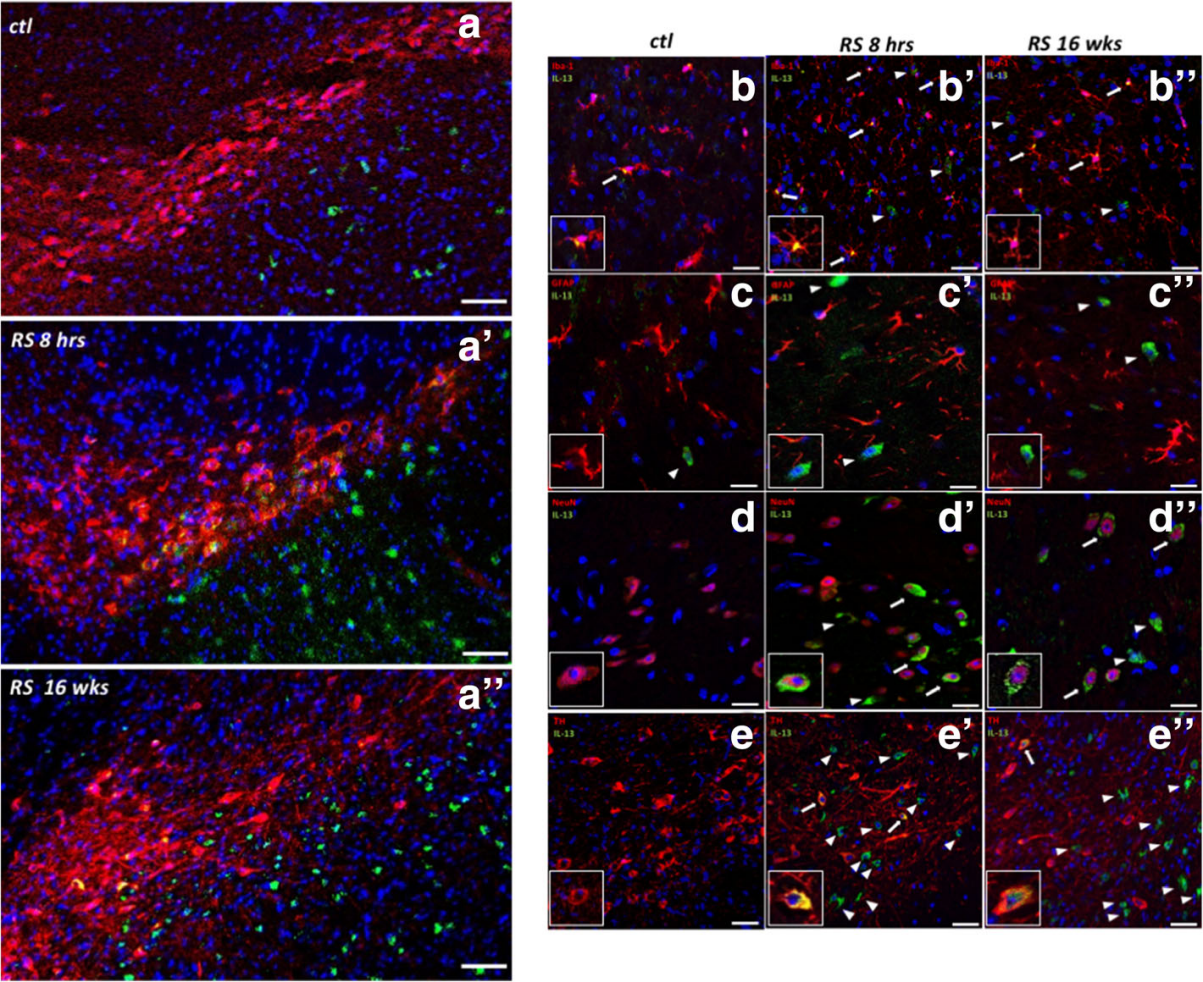
Mapping the location of IL-13 production in the substantia nigra. (**A**–**A**″) Representative pictures of immunofluorescent staining of IL-13 (*green*) in the SN of non-stressed (*top*), 8 h of RS (*center*) and after 16 weeks of RS (*bottom*); showing that RS elevates IL-13 expression in both the SNc and the SNr. (Pictures representative of a *n* = 3 experiment; *scale bars*: 0.5 mm; *blue*: DAPI). (**B**–**B**″, **C**–**C**″, **D**–**D**″, **E**–**E**″) Representative pictures of double immunofluorescence of IL-13 (*green*) with Iba-1, GFAP, NeuN, and TH (*red*), respectively, in control condition, 8 h of RS, and 16 weeks of RS. *Arrows*: co-localization of IL-13 and the specific cell type marker; *arrowheads*: IL-13 signal without co-localization with the cell type marker. IL-13 co-localizes (*arrows*) with microglia (Iba-1, Fig. **B**–**B**″), neurons (NeuN, Fig. **D**–**D**″), and dopaminergic cells (TH, Fig. **E**–**E**″). No co-staining was found in astrocytes (GFAP, Fig. **C**–**C**″). (*scale bar*: 200 μm in **A**–**A**″, 50 μm in **B**–**E**″; *blue*: DAPI)

Immunohistochemistry further confirmed that IL-13 protein expression increased at 8 h of RS and demonstrated that the cellular source of IL-13 was distributed in both the SN *pars compacta* (SNc) and the SN *pars reticulata* (SNr) (Fig. 3A–A′). Similar results were obtained when evaluating the presence and distribution of IL-13 by immunohistochemistry at 16 weeks of stress (Fig. 3A′′). These data indicate that stress paradigm used here elevated IL-13 providing evidence that the treatment used effectively led to a local increase of IL-13Rα1 ligand.

### In the substantia nigra IL-13 is produced by microglia and neurons

The source of IL-13 in the SN after acute and long-term RS exposure was determined by dual immunohistochemistry staining as well as by a combination of in situ hybridization/immunohistochemistry (Fig. 3 and Additional file 1: Figure S1C–C′′). Double immunofluorescence was performed for IL-13 and one of the following: the microglial marker Iba-1, the astrocytic marker GFAP, the pan-neuronal marker NeuN, or the catecholaminergic neuronal marker tyrosine hydroxylase (TH). IL-13 was expressed in the SNr and in part of the SNc (Fig. 3a) and co-localized with Iba1 as well as with NeuN (Fig. 3B–B′′, D–D′′) but not with GFAP (Fig. 3C–C′′). IL-13 was also found in a fraction of the TH-positive neurons in the SNc (Fig. 3E′–E′′). Following acute RS, microglia accounted for approximately 77% (4123 ± 204 Iba-1-positive cells out of 6450 ± 444 IL-13-positive cells) of the IL-13-positive cells. NeuN- and TH-positive cells accounted for 14 and 7%, respectively, of the remaining IL-13-positive cells. Similar values were detected after chronic stress exposure (Iba-1: 82%, NeuN: 13%, and TH: 4%), (see Additional file 1: Fig. S1B). Neuronal expression of IL-13 was also confirmed by double in situ hybridization/immunofluorescence for IL-13 mRNA and NeuN, respectively (see Additional file 1: Figure S1C–C″).

### Oxidative stress and microglia activation during restraint stress are similar in *Il13ra1*^*Y/−*^ and *Il13ra1*^*Y/+*^ mice

**Fig. 4:**
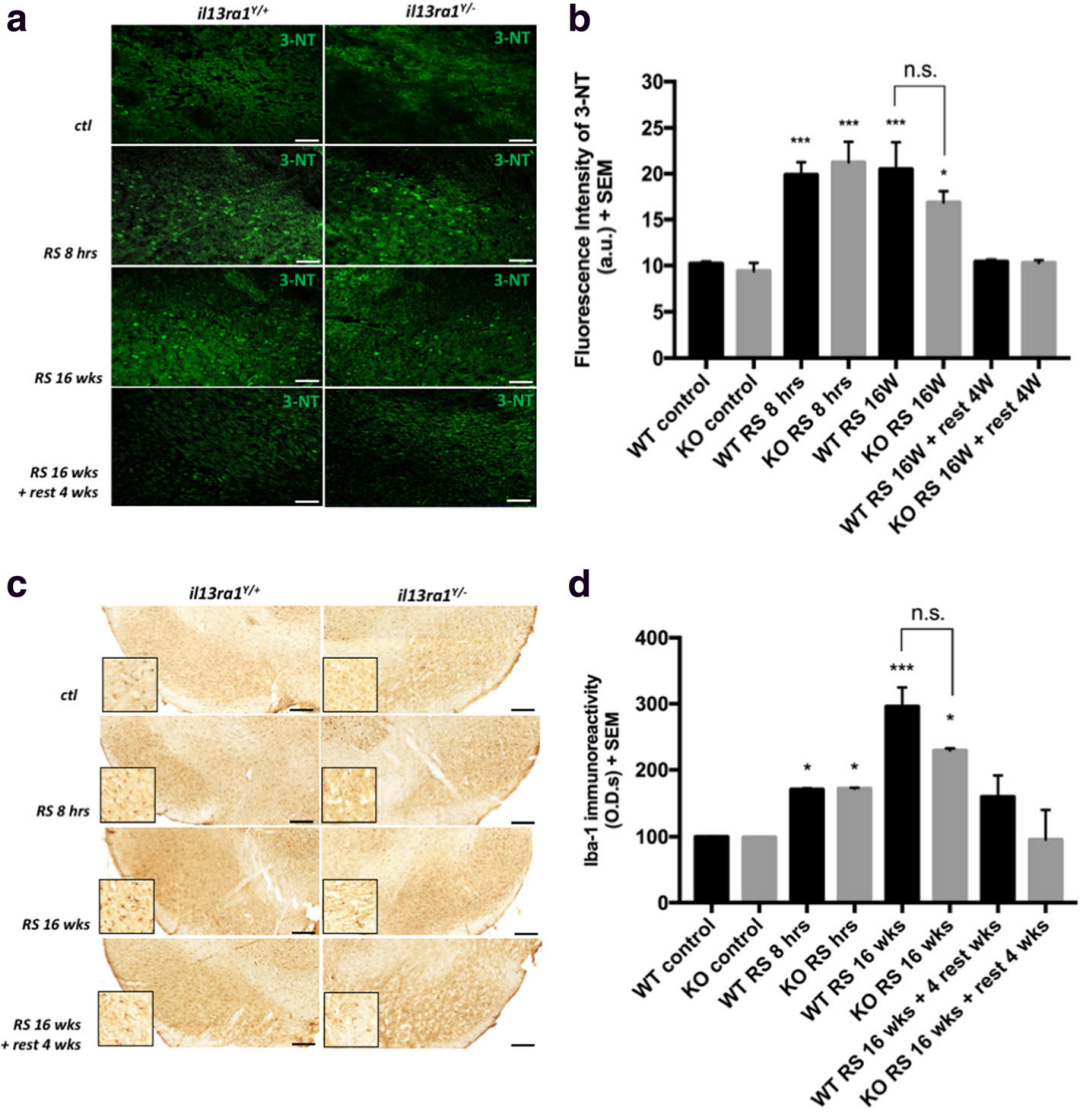
IL-13Rα1 does not alter restraint stress-associated neuroinflammation. **a** Representative images showing immunofluorescence of 3-Nitrotyrosine (3-NT, *green*) in the SN under resting conditions and at different paradigms of RS in IL-13Rα1 wild-type and knock-out animals. Levels of oxidative damage were increased during restraint stress with no difference between genotypes. **b** Histograms showing the average levels of fluorescence intensity (in a.u.) of 3-Nitrotyrosine fluorescence in the SN. **c** Representative pictures of microglial marker Iba-1 under resting conditions and at different paradigms of RS in IL-13Rα1 wild-type and knock-out animals. Levels of oxidative were increased during restraint stress with no difference between genotypes. **d** Histograms showing the average levels of O.D.s (in a.u.) of Iba-1 in the SN (*p < 0.05, ***p < 0.001 compared to control condition, *n* = 4–6; *scale bars*: 100 μm in A, 200 μm in **C**)

Activation of IL-13Rα1 contributes to neurotoxicity by increasing cellular susceptibility to oxidative stress [26]. We measured 3-Nitrotyrosine (3-NT) as an index of oxidation (Fig. 4a, b). We found that 3-NT levels were similar in both *Il13ra1* ^*Y/−*^ and *Il13ra1* ^*Y/+*^ non-stressed mice (9.42 ± 0.86 a.u. vs*.* 10.22 ± 0.23 a.u., *n* = 4, NS) and were increased similarly and significantly (*p* < 0.001) and in both groups following 8 h of RS (21.23 ± 2.24 a.u. vs*.* 19.89 ± 1.36 a.u.; *n* = 4–6, NS). At 16 weeks of RS, 3-NT levels across genotypes were still significantly higher than in non-stressed animals used as controls but did not differ statistically in *Il13ra1* ^*Y/−*^ compared to *Il13ra1* ^*Y/+*^ mice (16.86 ± 2.18 a.u. vs*.* 20.52 ± 2.91 a.u., *p* = 0.8). 3-NT levels at 20 weeks, 4 weeks after termination of stress, were similar to those of non-stressed controls animals and were not statistically different across genotypes (10.29 ± 2.18 a.u. vs. 10.43 ± 0.14 a.u., *n* = 4, NS). These data indicate that RS increases oxidative stress similarly in both genotypes demonstrating that ROS required for IL-13 toxicity were equally present in *Il13ra1* ^*Y/−*^ and *Il13ra1* ^*Y/+*^ mice.

Evaluation of the microglial marker Iba-1 at the same time points in *Il13ra1* ^*Y/−*^ and *Il13ra1* ^*Y/+*^ mice showed that RS increase microglia activation similarly in knock-out and wild-type animals (Fig. 4c, d). Iba-1 immunoreactivity was significantly increased in for *Il13ra1* ^*Y/−*^ mice and their wild-littermates after acute and chronic RS (*p* < 0.05 at 8 h and 16 weeks for *Il13ra1* ^*Y/−*^, *p* < 0.05 at 8 h, and *p* < 0.001 at 16 weeks for *Il13ra1* ^*Y/+*^ *)* and was lowered after 4 weeks of non-stressed condition (NS all groups). No statistical difference was detected at any time point between genotypes. These data confirm previous finding that RS induce neuroinflammation in the SN of wild-type animals and indicate that *Il13ra1* ^*Y/−*^ mice present the same level of microglial activation response after RS.

### Lack of interleukin-13 receptor α1 delays the loss of dopaminergic neurons during chronic stress

**Fig. 5:**
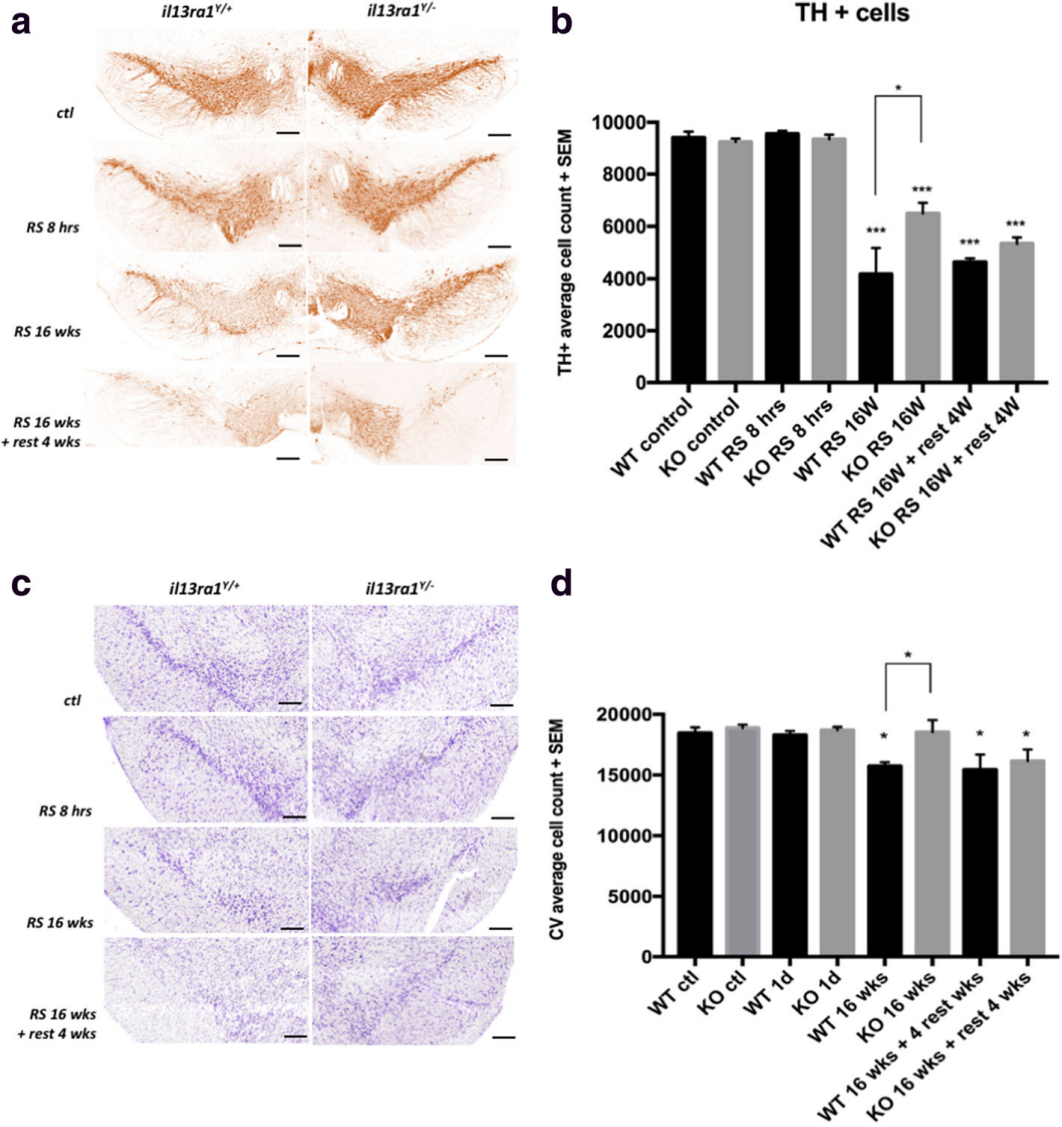
IL-13Rα1 contributes to dopaminergic neuronal loss during chronic stress. **a** Representative images of labeling of TH+ neurons using wild-type *Il13ra* ^*Y/+*^ and knock-out *Il13ra* ^*Y/−*^ mouse brains from control mice and at different time points in the RS paradigm. **b** Histograms showing the number of TH+ neurons in the *Il13ra* ^*Y/+*^and *Il13ra* ^*Y/−*^ in the SNc under control conditions, RS 8 h, after 16 weeks of RS, and after 16 weeks of RS plus 4 weeks of rest. **c** Representative images of cresyl violet labeling (Nissl staining) cells used in adjacent section to the ones assessed by TH staining. **d** Histograms showing the average cell count of CV+ cells in the *Il13ra* ^*Y/+*^ and *Il13ra* ^*Y/−*^ in the SNc under control conditions, RS 8 h, after 16 weeks of RS, and after 16 weeks of RS plus 4 weeks of rest. (*p < 0.05, ***p < 0.001 compared to control condition, *n* = 4–6; *scale bars*: 200 μm in **A**, **C**)

We next evaluated whether RS affected similarly or differently the number of DA neurons in *Il13ra1* ^*Y/−*^ and *Il13ra1* ^*Y/+*^ mice (Fig. 5a, b). Non-stressed *Il13ra1* ^*Y/−*^ and *Il13ra1* ^*Y/+*^ mice had similar numbers of TH-positive neurons in the SNc (9405 ± 231 for *Il13ra1* ^*Y/+*^ mice vs. 9240 ± 127 for *Il13ra1* ^*Y/−*^ mice, *n* = 4, NS), and these numbers were found to be similar following a single acute RS session (9556 ± 113 for *Il13ra1* ^*Y/+*^ mice vs. 9346 ± 176 for *Il13ra1* ^*Y/−*^ mice, *n* = 4, NS when compared to non-stressed control). Chronic RS reduced the number of TH-positive neurons in both wild-type *Il13ra1* ^*Y/+*^ (9405 ± 231 for control vs. 4180 ± 1001 for RS mice at 16 weeks, *n* = 4, *p* < 0.0001) and *Il13ra1* ^*Y/−*^ mice, (9240 ± 127 for control vs. 6490 ± 404 for RS mice, *n* = 4, *p* < 0.01). However, *Il13ra1* ^*Y/−*^ mice retained a significantly larger number of neurons than their wild-type littermates did (6490 ± 404 vs. 4180 ± 1001 for RS mice, *n* = 4, *p* < 0.05) indicating that *Il13ra1* ^*Y/−*^ mice are partially protected from the loss of DA neurons during chronic RS.

Measurements were also carried out on groups of animals that underwent the same stress paradigm but were analyzed following 4 weeks of rest after the 16 weeks of RS to evaluate whether the stress-dependent reduction in the number of TH-immunopositive cells would halt or continue. Data from these groups showed that the number of TH-positive neurons in the *Il13ra1* ^*Y/+*^ mice remained comparable to that observed at 16 weeks while the number of TH-positive neurons in the *Il13ra1* ^*Y/−*^ mice further decreased to reach a value similar to that in the *Il13ra1* ^*Y/+*^ mice (4647 ± 144 for *Il13ra1* ^*Y/+*^ mice vs. 5335 ± 239 for *Il13ra1* ^*Y/−*^, *n* = 4, NS). These data indicate that lack of IL-13Rα1 did not halt but rather delayed neuronal loss during RS. To ensure that the observed loss of TH immunoreactivity reflected cellular death rather than downregulation of TH expression, cell numbers were evaluated using cresyl violet staining on adjacent sections (Fig. 5c, d). We confirmed that the reduction of cells in the SN develops during chronic restraint stress in wild-type animals (*p* < 0.05 at 16 weeks and 16 weeks + rest compared to control) and is delayed in *Il13ra1* ^*Y/−*^ mice (*p* < 0.05 at 16 weeks compared to *Il13ra1* ^*Y/+*^ animals) but not completely halted where a statistical significant reduction was also found (*p* < 0.05 at 16 weeks + rest compared to control).

## Discussion

In this study, we tested the hypothesis that IL-13Rα1 is involved in the loss of DA neurons that is observed in a mouse model of severe chronic stress where animals are exposed to daily 8 h session of restraints stress for 5 consecutive days per week to a total of 16 weeks [11].

IL-13Rα1 is expressed on DA neurons of the SNc, and its activation was previously shown to increase the susceptibility of these cells to oxidative stress [26]. We found that the daily RS session elevated IL-13 in the substantia nigra, stimulated microglia activation, and elevated oxidative stress which is necessary for IL-13-mediated toxicity. We also collected evidence that IL-13 is produced in both the pars compacta (SNc) and the pars reticularis (SNr) regions of the substantia nigra, and that cellular sources comprises microglia and neurons, including a fraction of TH-positive ones. Neuronal expression of IL-13 was previously reported in the hippocampus and the cortex following an experimental ischemic insult [31, 32], and recent studies suggested a role of this cytokine in cognitive functions [33]. Our findings showed that stress can regulate IL-13 production locally and suggest the intriguing possibility that it may act in a paracrine or, in the case of TH neuron, possibly even in an autocrine fashion. We previously demonstrated that activation of IL-13Rα1 alone did not damage DA neurons indicating that the IL-13/IL-13Rα1 system is important for the physiology of dopaminergic cells [26]. Indeed, IL-13 was found to be toxic to dopaminergic cells of the SNc only in the presence of mild oxidative stress and, until now, only in experimental paradigms employing chronic prolonged insult like regular intermittent exposure to LPS for 6 months [26].

These features make the IL-13/IL-13Rα1 a particularly attractive subject of investigation in the etiology of PD, a neurodegenerative disease believed to develop and progress over a long period of time. For the same reason, we hypothesized that IL-13/IL-13Rα1 could have a role in the loss of dopaminergic neurons suggested to occur during chronic stress [7, 9, 11–13, 15, 34–39].

We previously showed the RS paradigm employed here (which is particularly severe and longer compared to other studies [40–43]) led to a 61% reduction in TH-positive neurons by 16 weeks of treatment [11], a finding recently replicated by an independent group using a similar paradigm [14]. Here, we further confirm the deleterious effects of chronic stress on DA neurons of the SNc as *Il13ra1* ^*Y/+*^ mice lost 56% of DA neurons in the SNc. We also found that mice lacking a functional IL-13Rα1 lost only 30% of cell. This, together with the evidence that the endogenous IL-13Rα1 ligand IL-13 level in the SN is elevated by restraint stress, suggests that IL-13 and IL-13Rα1 play a role in mediating the damaging effects of chronic stress on DA neurons. Stress induced oxidative damage and microglia activation, two possible mediators of neuronal damage, were affected similarly in *Il13ra1* ^*Y/−*^ and *Il13ra1* ^*Y/+*^ mice indicating that IL-13Rα1 did not alter these parameters. Therefore, it seems reasonable to conclude that one of the mechanisms accounting for the reduction in dopaminergic cell loss in the *Il13ra1* ^*Y/−*^ mice include the lack of the synergistic action between neuronal IL-13Rα1 and oxidative stress that was previously shown to exert toxicity in DA cell line [26].

In this study, we also addressed whether the damaging effects of chronic stress on dopaminergic neurons of the SNc continued upon termination of stress or not. We found that 4 weeks after stress was terminated, and the number of TH-positive neurons was similar to those measured at the termination of stress in *Il13ra1* ^*Y/+*^ but further decreased in *Il13ra1* ^*Y/−*^ to a number that was still higher but not statistically significant than that of wild-type mice. This indicates that the lack of IL-13Rα1 did not halt but rather delayed neuronal loss. The mechanisms accounting for such phenomenon remain to be determined. One possibility is that activation of IL-13Rα1 is only one of the factors contributing to the stress-dependent loss of these neurons. Another is that the stress paradigm utilized was so severe that the free radical damage caused develops over time to a level that is sufficiently large to damage even neurons lacking IL-13Rα1 overriding the neurotoxicity that is observed when both IL-13 and sub-lethal doses of reactive oxygen species are present. The use of stress as an experimental paradigm to investigate loss of DA neurons is at its infancy and it will be important to investigate the effects of shorter or milder stressors.

## Conclusions

We confirmed that severe chronic restraint stress can reduce the number of DA neurons and that the IL-13/IL-13Rα1 system is one, albeit not the only factor, contributing to these effects. We also showed that restraint stress might act as trigger of central production of IL-13. Blocking IL-13 or IL-13Rα1 may contribute to delay damage to DA neurons occurring during chronic severe stress.

## Additional files

Additional file 1: Methods: in situ hybridization. In situ hybridization was carried out as previously described [44] with fluorescent/peroxidase (POD) conjugate staining for IL-13. Anti-sense and sense digoxigenin (DIG)-labeled IL-13 riboprobes were synthesized using a commercial kit (Roche, Indianapolis, IN, USA) from a plasmid (pcr2.1-TOPO) containing full-length IL-13 cDNA (5′-CTT GCC TTG GTG GTC TCG-3′, 5′-CGT TGC ACA GGG GAG TCT-3′). Prehybridization and hybridization were then performed at 65 °C in a buffer containing 50% formamide, 2× SSC, 5× Denhardt’s reagent, 5% Dextrane sulfate, 0.5 mg/ml sheared salmon sperm DNA, and 0.25 mg/ml yeast total RNA. The probe was diluted in the hybridization buffer (800 ng/ml) and was incubated overnight on slides. After post-hybridization washes, slides were then blocked for 1 h and were incubated with anti-Digoxigenin-POD (11207733910 Roche), 1:1000 overnight at 4 °C. After several rinses in PBS-T, reactions were developed with TSA Plus Fluorescein substrate (NEL741001KT) for 10 min. Sections were then rinsed and were cover-slipped. Digital images were taken using a Zeiss LSM 710 laser scanning confocal microscope (LSCM). Figure S1 (A) Representative pictures of the region of interested evaluated in the cellular counting (substantia nigra, from −2.7 to −3.8 mm from bregma). (B) Graph showing double-labeled cells positive for IL-13 and for Iba-1, NeuN, or TH (*n* = 4, **p < 0.01 compared to control). (C–C″) In situ hybridization of IL-13 mRNA using anti-sense RNA probe in representative wt (*Il13ra* ^*Y/+*^) confirms co-localization with neuronal marker NeuN (*arrows*). Analysis with IL-13 sense probe yields no detectable signal (not shown). (Pictures representative of a *n* = 3 experiment, *scale bars*: 20 μm in G–G″, *blue*: DAPI). (TIFF 5303 kb)

## Abbreviations

3-NT: 3-Nitrotyrosine
CNS: Central nervous system
DA: Dopaminergic
IL-13: Interleukin-13
IL-13Rα1: Interleukin-13 receptor alpha 1
LPS: Lipopolysaccharide
LSCM: Laser scanning confocal microscope
MPTP: 1-methyl-4-phenyl-1,2,3,6-tetrahydropyridine
PD: Parkinson’s disease
ROS: Reactive oxygen species
RS: Restraint stress
SN: Substantia nigra
SNc: Substantia nigra pars compacta
TH: Tyrosine hydroxylase
