# Supplementary figures and images for: Lack of interleukin-13 receptor α1 delays the loss of dopaminergic neurons during chronic stress
Source: J Neuroinflammation. 2017 Apr 21;14:88. doi: 10.1186/s12974-017-0862-1 (PMC5399344; doi:10.1186/s12974-017-0862-1)

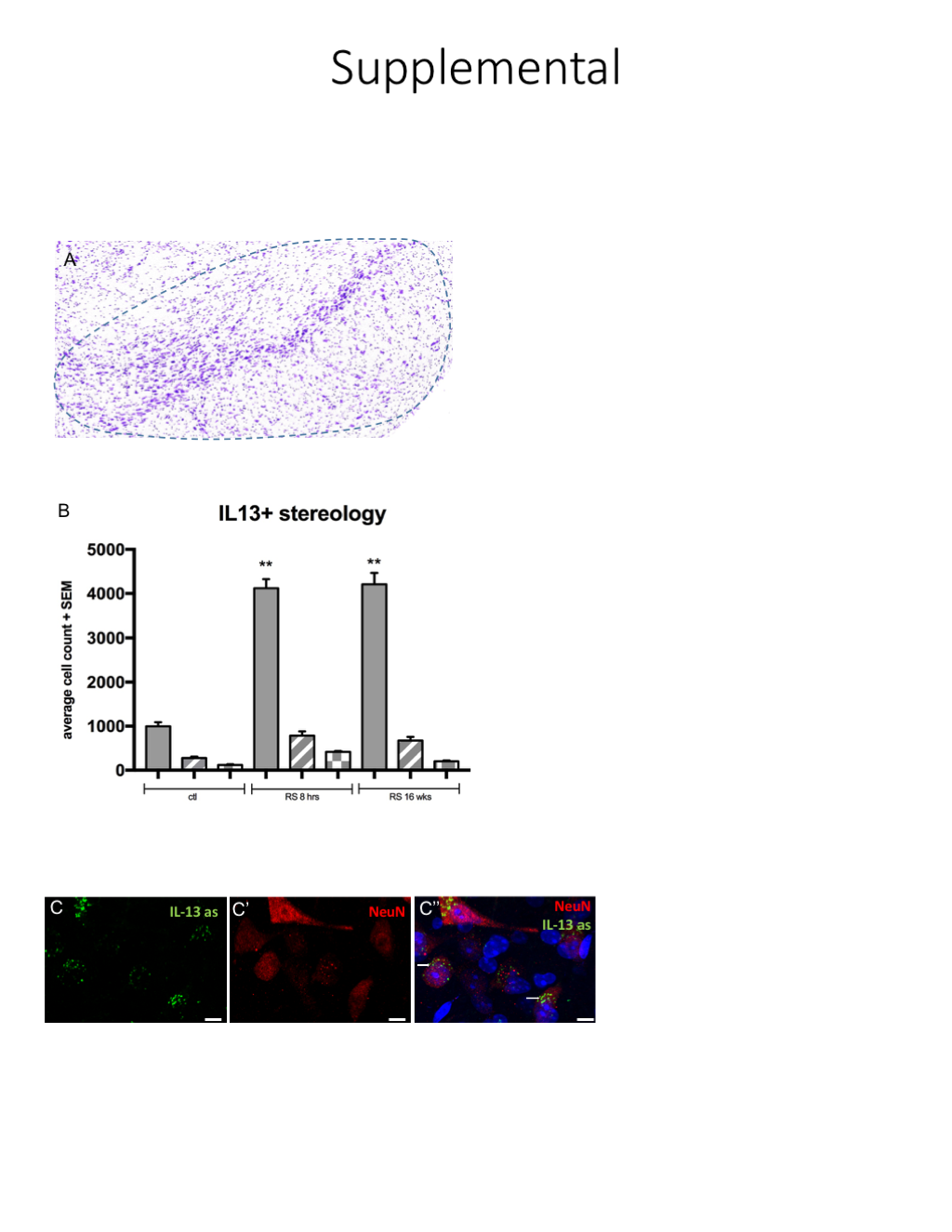

Supplement: Supplementary file 1 — Methods: in situ hybridization. In situ hybridization was carried out as previously described [44] with fluorescent/peroxidase (POD) conjugate staining for IL-13. Anti-sense and sense digoxigenin (DIG)-labeled IL-13 riboprobes were synthesized using a commercial kit (Roche, Indianapolis, IN, USA) from a plasmid (pcr2.1-TOPO) containing full-length IL-13 cDNA (5′-CTT GCC TTG GTG GTC TCG-3′, 5′-CGT TGC ACA GGG GAG TCT-3′). Prehybridization and hybridization were then performed at 65 °C in a buffer containing 50% formamide, 2× SSC, 5× Denhardt’s reagent, 5% Dextrane sulfate, 0.5 mg/ml sheared salmon sperm DNA, and 0.25 mg/ml yeast total RNA. The probe was diluted in the hybridization buffer (800 ng/ml) and was incubated overnight on slides. After post-hybridization washes, slides were then blocked for 1 h and were incubated with anti-Digoxigenin-POD (11207733910 Roche), 1:1000 overnight at 4 °C. After several rinses in PBS-T, reactions were developed with TSA Plus Fluorescein substrate (NEL741001KT) for 10 min. Sections were then rinsed and were cover-slipped. Digital images were taken using a Zeiss LSM 710 laser scanning confocal microscope (LSCM). Figure S1 (A) Representative pictures of the region of interested evaluated in the cellular counting (substantia nigra, from −2.7 to −3.8 mm from bregma). (B) Graph showing double-labeled cells positive for IL-13 and for Iba-1, NeuN, or TH (n = 4, **p < 0.01 compared to control). (C–C″) In situ hybridization of IL-13 mRNA using anti-sense RNA probe in representative wt (Il13ra Y/+) confirms co-localization with neuronal marker NeuN (arrows). Analysis with IL-13 sense probe yields no detectable signal (not shown). (Pictures representative of a n = 3 experiment, scale bars: 20 μm in G–G″, blue: DAPI). (TIFF 5303 kb) [file 12974_2017_862_MOESM1_ESM.tiff]
